# Toll-Interleukin 1 Receptor Domain-Containing Adaptor Protein 180L Single-Nucleotide Polymorphism Is Associated With Susceptibility to Recurrent Pneumococcal Lower Respiratory Tract Infections in Children

**DOI:** 10.3389/fimmu.2018.01780

**Published:** 2018-08-07

**Authors:** Johan N. Siebert, Lutz Hamann, Charlotte M. Verolet, Cécile Gameiro, Stéphane Grillet, Claire-Anne Siegrist, Klara M. Posfay-Barbe

**Affiliations:** ^1^Department of Pediatric Emergency Medicine, Geneva Children’s Hospital, Geneva University Hospitals, Geneva, Switzerland; ^2^Department of Pathology-Immunology and Pediatrics, Center for Vaccinology and Neonatal Immunology, Geneva Medical Center, Geneva, Switzerland; ^3^Institute of Medical Microbiology and Hygiene, Charité-University Medical Center Berlin, Berlin, Germany; ^4^Department of Pediatrics, Geneva Children’s Hospital, Geneva University Hospitals, Geneva, Switzerland; ^5^Flow Cytometry Core Facility, Geneva University Medical Center, Geneva, Switzerland; ^6^Faculty of Medicine, University of Geneva, Geneva, Switzerland

**Keywords:** toll-like receptors, *Streptococcus pneumoniae*, child, pneumonia, immunity, innate, polymorphism, single nucleotide

## Abstract

Lower respiratory tract infections (LRTI) are often caused by *Streptococcus pneumoniae* (*Spn*) and can be recurrent in 8% of children older than 2 years of age. *Spn* is recognized by pattern-recognition receptors (PRRs) of the innate immune system, in particular toll-like receptors (TLRs) 2 and 4. To assess whether a defect somewhere along this TLR signaling pathway increases susceptibility to recurrent pneumococcal LRTI, we conducted a prospective case–control study with 88 healthy individuals and 45 children with recurrent LRTI aged 2–5 years old. We examined cell surface expression of *TLR2* and *TLR4*, as well as eight genetic variants of these receptors or associated co-receptors TLR1 and TLR6. Interleukin-6 production was measured after whole blood stimulation assays with specific agonists and heat-killed *Spn*. Our findings reveal that single-nucleotide polymorphisms within toll-interleukin 1 receptor domain-containing adaptor protein (*TIRAP*) alone or in combination with TLR1 N248S, TLR1 I602S, or TLR6 S249P polymorphisms contributes to various degree of susceptibility to recurrent pneumococcal LRTI in children by modulating the inflammatory response. In that respect, carriage of the TIRAP S180L heterozygous trait increases the likelihood to protect against pneumococcal LRTI, whereas children carrying the mutant homozygous TIRAP 180L polymorphism might be more likely susceptible to recurrent pneumococcal LRTI.

## Introduction

Lower respiratory tract infections (LRTI), such as pneumonia and bronchitis are major causes of hospitalization in young children less than 5 years old, and are associated with significant morbidity and mortality (1, 2). *Streptococcus pneumoniae* (*Spn*) is the most common bacterial cause of community-acquired pneumonia (CAP) in childhood (3–6). Despite an early and high prevalence of nasopharyngeal pneumococcal colonization of the human upper respiratory tract, the immature immune system of young children responds poorly to the polysaccharidic antigens of *Spn* (7). This is reflected by an incidence of invasive pneumococcal disease (IPD) as high in young infants as in the elderly (8). After the second year of life, *Spn* infections become much rarer, which theoretically reflects immune maturation (8). However, approximately 8% of children older than 2 years of age hospitalized for CAP are recognized as having recurrent LRTI (9), of which a significant proportion is caused by *Spn* (10–14). While most children with recurrent infections probably have a normal immunity, it is important to recognize, and treat appropriately those with an underlying transient or permanent immune dysfunction.

The innate immune system is crucial for the control of colonization and for early recognition of invasive microorganisms to initiate an immediate protective response (15). *Spn* recognition starts at the cell surface of human monocytes through the pattern-recognition receptors (PRRs) family. Among them, the transmembrane glycoproteins toll-like receptors (TLRs) play a pivotal role (16). Upon this recognition, the TLRs regulates downstream intracellular MyD88-dependent and TRIF-dependent signaling pathways involving several adaptor proteins, including toll-interleukin 1 receptor domain-containing adaptor protein (*TIRAP*) (17). This activates transcription factors and results in the production of a diverse array of inflammatory mediators, such as interleukin-1 (IL-1), interleukin-6 (IL-6), and type I interferon. Previous studies have shown that TLR signaling defects can impair the innate immune responses and are associated with greatly enhanced susceptibility to invasive life-threatening infections (18–21). However, to the best of our knowledge, this has not been studied in young children with recurrent pneumococcal LRTI.

In this study, we investigate whether an abnormal or delayed maturation pattern of the innate immune response to pneumococcal pathogen-associated molecular patterns was present in young children with recurrent LRTI along the TLR signaling pathways. To achieve this, we explore the *TLR2* and *TLR4* monocytes’ cell surface expressions that are mainly involved in the host immune response against *Spn* (16). We also examine the influence of loss-of-function genetic variants in eight single and combined nucleotide polymorphisms (SNPs) in the corresponding TLRs, or their associated co-receptors, as well as in microRNA-146 reported to be a regulator of *TLR4* expression and inflammatory response (22). The findings are then correlated with functional assays investigating the SNP’s influence on the individual’s ability to produce an inflammatory response through IL-6 production after whole blood stimulation by specific *TLR2* and *TLR4* ligands. We determine their role in susceptibility to recurrent pneumococcal pneumonia in children.

## Materials and Methods

This prospective case–control study was conducted in a tertiary hospital with 30,000 outpatient consultations per year. The study was approved by the institutional ethics committee, and written consent was obtained from all parents or legal guardians. It was conducted in accordance with the principles of the Declaration of Helsinki, the standards of Good Clinical Practice, and Swiss regulatory requirements. Patients’ records were anonymized and de-identified before analysis. Health-related information, genetic data, and biological materials were stored in secured places at the Geneva University Medical Center and Geneva University Hospitals. They were at the disposition of the investigators for the sole purpose of the study. Only small samples of blood consistent with physiological minimal risk were harvested from children. Residual blood was destroyed.

### Patients and Healthy Subjects

The patients were previously described (14). Briefly, cases were children between 2 and 5 years of age with recurrent LRTI, defined as having had two or more episodes of pneumonia in a single year or at least three episodes (12). The initial diagnosis had to be either confirmed clinically according to the WHO criteria (23) and/or by radiology, with complete clinical resolution between occurrences. Patients were referred through their pediatrician, the infectious diseases or the pulmonology outpatient clinic, the laboratory of vaccinology, or recruited at time of hospitalization for repeated pneumonia. Exclusion criteria were as follows: anatomic anomalies of the respiratory tract, primary ciliary dyskinesia, cystic fibrosis, foreign body in the respiratory tract, asthma or severe atopic disease, known congenital or acquired immunodeficiencies, lymphopenia, or intravenous immunoglobulin substitution therapy. Cases were followed prospectively and had blood samples on a yearly basis. Samples were taken outside of acute infectious episodes to avoid measurement of related cellular changes or inflammation. Healthy controls were age-matched children referred for elective surgery not related to the lungs, who provided a single blood sample at enrollment. They had no history of LRTI and had been immunized against *Spn*, in accordance with the Swiss recommendations.

### Blood Sampling and Leukocytes Isolation

Fresh peripheral blood (PB) samples were placed into sterile heparinized-tubes (BD Vacutainer, Becton Dickinson, Franklin Lakes, NJ, USA) and processed on the day of collection. Plasma was isolated by centrifugation (1,500 rpm, 5 min, 4°C) and stored at −80°C. Twenty microliters of naïve mouse serum were added to 100 µl of whole blood to prevent Fc-mediated interactions, and incubated at room temperature (RT), in the dark. After 5 min, 20 µl of isotype control, anti-*TLR2*, or anti-*TLR4* monoclonal antibodies was added to the blood samples. After five additional minutes, 20 µl of anti-CD14 monoclonal antibodies was added to each sample and incubated for another 20 min, at RT in the dark. Then, cells were washed once with PBS 0.1% sodium azide and centrifuged (1,500 rpm, 5 min, RT). Supernatant was decanted, and the contaminating erythrocytes were lysed by resuspending the cell pellet with 3 ml pH 7.4 BD Pharm Lyse™ lysing solution diluted in sterile pyrogen-free H_2_O (1:10). The samples were incubated 15 min, in the dark, at RT followed by washing twice with FACS Buffer (prepared by adding 50 µl NaN_3_ + 0.5 g BSA to 50 ml PBS 0.1% sodium azide) and centrifuged (1,500 rpm, 5 min, 4°C). Supernatant was decanted, and the cell pellet resuspended in 100 µl of FACS buffer and transferred into sterile Falcon 96-well Microplate U-bottom (BD Biosciences, VWR, Switzerland). Finally, the plate was centrifuged, supernatant was decanted, and the cell pellet resuspended by 120 µl of FACS buffer before going to the cytofluorometer. All experiments were conducted on the day of blood collection.

### Flow Cytometry Analysis

PBMC were stained with appropriate combinations of phycoerythrin (PE) and allophycocyanin (APC) monoclonal antibodies. Optimal working dilutions of the antibodies were assessed in titration assays. PE anti-human *TLR2* mAb (clone TL2.1, mouse IgG2a κ, RRID:AB_10667887), PE anti-human *TLR4* mAb (clone HTA125, mouse IgG2a κ, RRID:AB_1603311), PE isotype control (clone eBM2a, mouse IgG2a κ, RRID:AB_470063), and APC anti-human CD14 mAb (clone 61D3, mouse IgG1, RRID:AB_10669167) were all purchased from eBioscience (THP Medical Products, Switzerland). Cells were all analyzed using a fluorescence-activated cell sorting FACSArray flow cytometer (BD Biosciences, San Diego, CA, USA) interfaced with BD FACSDIVA™ software (v.6.1.3, BD Biosciences). Side/forward scatter dot plot was used to exclude dead cells and to gate the live monocyte population. To refine the recognition of TLR expression on monocytes, double staining for CD14 and *TLR2*, CD14 and *TLR4*, or CD14 and IgG2a control isotype was used. The expression of these surface markers was calculated as median fluorescence intensity (MFI) in non-overlapping Yellow-A (PE fluorochrome) and Red-A channels (APC fluorochrome) and recorded for each patient.

We also used flow cytometry in a pilot experiment to determinate which pro-inflammatory cytokine would best reflect the TLR-induced inflammatory response following whole blood stimulation assay. Thanks to a multiplex cytometric bead array (CBA) kit (Human Inflammation Kit; BD Biosciences Pharmingen, San Diego, CA, USA), we simultaneously quantified IL-6, IL-8, IL-1β, IL-10, IL-12 p70, and tumor necrosis factor-α (TNF-α) production. This CBA kit consists of a mix of six microbead populations dyed to six different fluorescence intensities. Each microbead is coated with a monoclonal antibody against a given cytokine. Following incubation with the test sample, the bead-captured cytokines are simultaneously detected and quantified by direct immunoassay using six different antibodies labeled with phycoerythrin (PE). Our test sample was supernatant collected after centrifugation (1,000 rpm, 5 min, 4°C) of healthy human whole blood previously stimulated and incubated during 6 h with Ultrapure *E. coli* K12 LPS (LabForce, Switzerland). We found that IL-6 was the fastest and most discriminative cytokine in the supernatant. The experiment was performed according to the manufacturer’s instructions using the 4-colors FACSArray flow cytometer. Standard curves were generated for each cytokine using the standard provided in the kit. Each cytokine concentration within the supernatant was interpolated from these standard curves.

### *In Vitro* Whole Blood Functional Assays

We performed measurements of cellular *in vitro* responses to four TLR agonists and to *Spn* in whole blood functional assays. It has been shown that TLR function, as assessed by the production of IL-6, is stable between birth and 60 years old (24) and that whole blood is the best way to study *in vitro* cytokine production (25). Because these are more strongly correlated with monocytes than cytokines from purified PBMC (26). In most studies, however, *in vitro* cytokine production has been studied with mononuclear cells isolated from PBMC. This method suppresses the complex physiological interactions normally expected between immune cells and mediators and does not faithfully reflect the natural milieu (24). All procedures were performed in sterile conditions using sterile instruments and fluids. Fresh PB samples were obtained and placed into heparinized syringes. On collection day (maximum 1 h delay), blood was diluted 1:2 in a reservoir (Corning Life Science Costar^®^ reagent reservoirs, Vitaris AG, Switzerland) with heparinized culture medium RPMI 1640 with GlutaMAX™ I, 25 mM HEPES (Invitrogen, Switzerland) pre-treated with 50 µg/ml gentamicin (Sigma-Aldrich, Switzerland). Blood was plated at 60 µl/well in a 96-well U-bottom microplate (Nunc, Milian, Switzerland) and incubated at 37°C for 15 min with 5% CO_2_. The following TLR agonists were used at optimal concentrations that elicited cytokine responses as preliminarily determined by dose–response curves: (1) Ultrapure *E. coli* K12 LPS (200 ng/ml final), (2) Pam_3_CSK_4_ (10 µg/ml final), and (3) Muramyl Dipeptide (MDP, 200 µg/ml final) were all purchased from InvivoGen (LabForce, Switzerland). MDP (200 µg/ml final), which is recognized by the nucleotide-binding oligomerization domain (NOD2) protein immune receptor, was also used in combination with Pam_3_CSK_4_ (20 µg/ml final). (4) TLA4e/AF04 (400 ng/ml final) was kindly provided by Sanofi Pasteur. TLA4e is constituted with an oil-in-water emulsion as described in WO 07/006939 and the Eisai product ER 804057 (also known as E6020, described in US 7,683,200) which is a *TLR4* agonist. In addition, 1 × 10^8^ cfu/ml heat-killed *Spn* were used (see below). Each agonist was serially diluted 1:2 (eight dilutions available) with filtered (Steriflip Millipore 0.22 µm, Milian, Switzerland) RPMI 1640 supplemented with 0.25% human serum albumin 20% (CSL Behring AG, Switzerland), and 60 µl of each sample was added 1:2 to individual blood wells in duplicate. Non-stimulated blood (with RPMI alone) was used to measure the unspecific negative control. After 6 h of incubation at 37°C with 5% CO_2_, supernatants were collected after centrifugation (1,000 rpm, 5 min, 4°C), and IL-6 was measured by enzyme-linked immunosorbent assay (ELISA).

### Preparation of Heat-Killed Spn

*Streptococcus pneumoniae*, serotype 22, was obtained from our hospital’s bacterial culture collection. Modified from a previous protocol (27), colonies were incubated overnight at 37°C on a Columbia AGAR (sheep blood 5%) plate with 5% CO_2_. Colonies were used to inoculate 10 ml of brain heart infusion (without AGAR). The inoculum was incubated for 16 h at 37°C and added to 1,000 ml of the same media. Bacteria were grown to logarithmic phase growth. To confirm that the bacterial growth has reached log phase, we used a spectrometer and read the culture broth at 600 nm to fall between OD_600_ = 0.4–0.6. Bacteria were stopped from growing further by incubating at 4°C. After centrifugation at 6,000 *g* for 10 min at 4°C, the entire bacteria pellet was washed two times with PBS and killed by incubation at 70°C for 1 h. Killing was confirmed by cultivation on sheep blood agar plates, and the presence of *Spn* was confirmed by microscopic examination and optochin inhibition test. Bacteria were stored at −80°C in 20% glycerol/80% PBS.

### IL-6 Enzyme-Linked Immunosorbent Assay

Interleukin-6 was measured by performing an ELISA using 96-well flat-bottom plates (Nunc Maxisorp™, Milian, Switzerland), previously coated with 1.01 µg/ml purified mouse anti-human IL-6 (clone 5IL6, IgG1, κ) mAb (Thermo Fisher Scientific, Perbio Science, Switzerland). On the day of experiments, plates were washed four times with PBS/0.05% Tween 20 and then saturated with PBS/0.05% Tween 20/16% human serum albumin (Sigma-Aldrich, Switzerland) during 1 h at 37°C. Then, 0.5 µg/ml of mouse anti-human IL-6 biotinylated (clone 7IL6, IgG1) mAb (Thermo Fisher Scientific, Perbio Science, Switzerland) was added in each well (50 µl/well). The first International standard IL-6 reference (NIBSC no. 89/548) was added into the plate by using serial dilutions 1:2 (from 20 ng/ml to 156 pg/ml final, 50 µl/well). Supernatants obtained after whole blood stimulation assays were added to the remaining wells (50 µl/well), and the samples were incubated 2 h at 37°C in the dark. Two blank columns served as negative controls. Plates were then washed four times with PBS/0.05% Tween 20 and incubated 1 h at 37°C with 1:1,000 ExtrAvidin 50 µl/well (Sigma-Aldrich, Switzerland). Plates were again washed four times and were developed using ABTS (2,2′-azino-bis[3-ethylbenzothiazoline-6-sulfonic acid]-diammonium salt) as a substrate, plus 10 μl H_2_O_2_ after 30 min of incubation. The optical density was determined at a wavelength of 405 nm using a Spectramax plate reader interfaced with a SoftMax Pro software (Molecular Devices, Sunnyvale, CA, USA). All values were interpolated based on the standard IL-6 curve. IL-6 concentrations were expressed as picograms per milliliter and normalized for 10^4^ monocytes (monocytes blood count was determined by flow cytometry); the values were logarithmically (log_10_) transformed.

### DNA Preparation and Genotyping

Genomic DNA was prepared by standard procedures from whole blood. Genotyping for *TLR1* (rs4833095, rs5743618), TLR*2* (rs5743708), *TLR4* (rs4986790, rs4986791), *TLR6* (rs5743810), *TIRAP* (rs8177374), and miR-164a (rs2910164) was performed by PCR including fluorescence-labeled hybridization FRET probes followed by melting curve analysis employing the LightCycler 480 (Roche Diagnostics). Primer and probes used in this study are shown in Table 1. Details have been published previously (28–33).

**Table 1:** Primers and probes used in this study.

TLR1, SNP ID: 1. rs4833095 and 2. rs5743618	
TRL1-forward-primer-1	ttggatgtgtcagtcaagactgtag
TRL1-reverse-primer-1	gcttcacgtttgaaattgag
TRL1-anchor-probe-1	LC-Red640-gtttgaagtttcgccagaatacttagg
TRL1-sensor-probe-1	ttaaggtaagacttgataactttgg-FL
TRL1-forward-primer-2	tgtgactacccggaaagttataga
TRL1-reverse-primer-2	cccagaaagaatcgtgcc
TRL1-anchor-probe-2	LC-Red640-cctccctctgcatctacttggat
TRL1-sensor-probe-2	ccatgctggtgttggctgtgactgtg-FL

**TLR2, SNP ID: rs5743708**

TRL2-forward-primer	tcccatttccgtctttttga
TRL2-reverse-primer	aggactttatcgcagctctcag
TRL2-anchor-probe	LC-Red640-cctacctggagtggcccatggacg
TRL2-sensor-probe	caagctgcagaagataatgaacaccaag-FL

**TLR4, SNP ID: 1. rs4986790 and 2. rs4986791**

TRL4-forward-primer-1	atttaaagaaattaggcttcataagct
TRL4-reverse-primer-1	ccaagaa-gtttgaactcatggtaa
TRL4-anchor-probe-1	LC-Red640-aattgtttgacaaatgtttcttcattttcc
TRL4-sensor-probe-1	ctactacctcgatgatattattgacttatt-FL
TRL4-forward-primer-2	atttaaagaaattaggcttcataagct
TRL4-reverse-primer-2	ccaagaagtttgaactcatggtaa
TRL4-anchor-probe-2	LC-Red640-attttgggacaaccagcctaaagtat
TRL4-sensor-probe-2	cttgagtttcaaaggttgctgttctcaaagt-FL

**TLR6, SNP ID: rs5743810**

TRL6-forward-primer	gaaagactctgaccaggcat
TRL6-reverse-primer	ctagtttattcgctatccaagtg
TRL6-anchor-probe	LC-Red640-ttaccctcaaccacatagaaacgacttgga
TRL6-sensor-probe	accagaggtccaaccttactgaa-FL

**TIRAP, SNP ID: rs8177374**

TIRAP-forward-primer	gccaggcactgagcagtagt
TIRAP-reverse-primer	gtgggtaggcagctcttctg
TIRAP-anchor-probe	LC-Red640-gatggtgcagccctcggcccc
TIRAP-sensor-probe	aggcccaacagcaggg-FL

**miR-164a, SNP ID: rs2910164**

miR-164a-forward-primer	ccatctctgaaaagccgatgtgta
miR-164a-reverse-primer	ggatctactctctccaggtcctcaag
miR-164a-anchor-probe	LC-Red 640-tcttcagctgggatatctctgtcatcgt
miR-164a-sensor-probe	tcagtgtcagacctctgaaattcag-FL

*SNP, single-nucleotide polymorphism; FL, fluorescein; LC-Red640, LightCycler Red-640*.

### Statistical Analysis

Data were displayed as arithmetic mean and 95% CI or median and interquartile ranges (IQRs). Hardy–Weinberg equilibrium was examined using a goodness-of-fit χ^2^ test with 1 degree of freedom to compare the observed and expected genotype distributions among the study subjects. Association between SNPs and risk of recurrent LRTI were tested using four common genetic models of inheritance (codominant, dominant, recessive, and overdominant) analysis by multiple logistic regressions to obtain odds ratios (ORs), 95% confidence intervals (CIs), and *P*-values. Codominant1 [homozygous wild type (WT) versus heterozygous (HT)], codominant2 (WT versus homozygous mutant (HM)), codominant3 (HT versus HM), dominant (WT versus HM + HT), recessive (HM versus WT + HT), and overdominant (HT versus WT + HM) models were assessed (34). All variables with a *P*-value lower than 0.2 in the univariate models were used in multivariate logistic models to adjust our result for gender and origin. Akaike’s Information Criterion was applied to estimate the best-fit model for each SNP; the model with the least AIC value was the most probable. Pairwise linkage disequilibrium (LD), *D*′, and Pearson’s *r* correlation were calculated using a web-based software for SNP analysis (http://bioinfo.iconcologia.net/SNPstats/). Based on associated biological TLR pathways and on the results of the single gene associations, we also tested the joint effects of combinations of SNPs pairs. For group comparisons, Student’s *t*-test or Wilcoxon test was applied to compare continuous variables, depending on their distribution. The Anderson–Darling test for normality was used to assess the IL-6 distribution. Chi-square and Fisher’s exact tests were used for dichotomic variables. Linear regressions were used to analyze associations between variables. Data analyses were performed on STATA (version IC 14.0; StataCorp LP, College Station, TX, USA).

## Results

### Patient’s Characteristics

Forty-five children with recurrent pneumonia and 88 healthy controls aged 2–5 years were enrolled. The mean age (SD) was 42.7 (13.0) and 44.3 (11.9) months, respectively (*P* = NS). Age categories were arbitrarily distributed in two groups: group 1 (24–48 months) and group 2 (48–72 months). Among the 45 LRTI children, 24 were males and 21 were females. Among the 88 healthy children, there was a gender predominance of 54 males over 34 females. This predominance was the result of a selection bias (elective surgery for circumcision). Ethnicities were equally distributed among children, with 76% of LRTI children and 73% of healthy children being Caucasians. Among the remaining LRTI children, 6 (13%) were of mixed origins, 2 (4%) were Middle Eastern, 2 (4%) were South American and 1 (2%) was from Sri Lanka.

### Hardy–Weinberg Equilibrium

Genotype distributions of *TLR1* (rs4833095 and rs5743618), *TLR2* (rs5743708), *TLR4* (rs4986790 and rs4986791), *TLR6* (rs5743810), and *miR-146a* (rs2910164) SNPs were consistent with Hardy–Weinberg equilibrium among both healthy and LRTI children (data not shown). *TIRAP* (rs8177374) SNP was not in Hardy–Weinberg equilibrium, both in LRTI and healthy children (*P* < 0.0001).

### TLR Genotyping Reveals Homozygous TIRAP S180L Polymorphism Predominance Traits in LRTI Children

Wild-type (WT), heterozygous (HT), and homozygous (HM) traits were compared between healthy and LRTI children for eight SNPs. The results are summarized in Table 2. The differences in the number of patients screened for SNPs either in healthy or LRTI children were due to not usable DNA. Compared with WT, carriage of the *TIRAP* heterozygous trait in the codominant model was higher in the healthy population than in LRTI children (24.7 versus 7.3%, respectively, OR 0.27 [95% CI 0.07–1.01], *P* = 0.03). Compared with HM, carriage of the *TIRAP* HT trait was also predominantly observed in the healthy children (OR 0.18 [95% CI 0.045–0.74], *P* = 0.017). This association was also seen in the overdominant model, where the HT trait was more observed in the healthy population (*P* = 0.013). On the other hand, in the codominant model, the HM trait was more observed in the LRTI population when compared with HT (OR 5.44 [95% CI 1.35–22.01], *P* = 0.017). When compared with WT, carriage of the *TIRAP* HM trait was also higher in the LRTI children but without reaching significance (*P* = 0.36). No associations were observed between all the other single SNPs studied and recurrent pneumococcal LRTI. Neither gender nor origins have shown any influence on the results. The allelic frequencies compared between both healthy and LRTI children were not significant.

**Table 2:** Relationship between TLR single-nucleotide polymorphisms and recurrent LRTI in children.

		Genotype frequencies						
SNP	Genotype	Healthy, *n* (%)	LRTI, *n* (%)	Genetic model	OR^a^	95% CI^a^		Adjusted *P*-value (χ^2^)^b^
TLR1 rs4833095	CC	32 (36.4)	12 (26.7)	Codominant1	1.60	0.68–3.77		0.52
	CT	35 (39.8)	21 (46.7)	Codominant2	1.52	0.58–4.02		
	TT	21 (23.9)	12 (26.7)	Codominant3	0.95	0.39–2.32	
				Dominant^c^	1.57	0.71–3.46		0.26
				Recessive	1.16	0.51–2.64		0.72
				Overdominant	1.32	0.64–2.74		0.45

TLR1 rs5743618	GG	19 (21.6)	9 (20)	Codominant1	0.97	0.37–2.57		0.80
	GT	39 (44.3)	18 (40)	Codominant2	1.27	0.47–3.39		
	TT	30 (34.1)	18 (40)	Codominant3	1.30	0.58–2.92		
				Dominant	1.10	0.45–2.68		0.83
				Recessive^c^	1.29	0.61–2.71		0.50
				Overdominant	0.84	0.40–1.74		0.63

TLR2 rs5743708	GG	81 (92)	43 (95.6)	Codominant1	0.54	0.11–2.70		0.43
	GA	7 (8)	2 (4.4)	Codominant2	NA	NA		
	AA	0	0	Codominant3	NA	NA		
				Dominant	NA	NA		
				Recessive	NA	NA		
				Overdominant	NA	NA		

TLR4 rs4986790	AA	69 (79.4)	41 (91.1)	Codominant1	0.40	0.12–1.26		0.16
	AG	17 (19.5)	4 (8.9)	Codominant2	0.00	0.00–NA		
	GG	1 (1.1)	0	Codominant3	0.00	0.00–NA		
				Dominant^c^	0.37	0.12–1.18		0.07
				Recessive	0.00	0.00–NA		0.36
				Overdominant	0.40	0.13–1.28		0.10

TLR4 rs4986791	CC	69 (79.3)	41 (91.1)	Codominant1	0.37	0.12–1.18		0.07
	CT	18 (20.7)	4 (8.9)	Codominant2	NA	NA		
	TT	0	0	Codominant3	NA	NA		
				Dominant	NA	NA		
				Recessive	NA	NA		
				Overdominant	NA	NA		

TLR6 rs5743810	TT	46 (52.3)	21 (46.7)	Codominant1	1.10	0.50–2.38		0.54
	TC	34 (38.6)	17 (37.8)	Codominant2	1.92	0.61–5.98		
	CC	8 (9.1)	7 (15.6)	Codominant3	1.75	0.54–5.64		
				Dominant	1.25	0.61–2.57		0.54
				Recessive^c^	1.84	0.62–5.45		0.27
				Overdominant	0.96	0.46–2.02		0.92

TIRAP rs8177374	CC	46 (54.1)	24 (58.5)	Codominant1	0.27	0.07–1.01		0.03*
	CT	21 (24.7)	3 (7.3)	Codominant2	1.49	0.63–3.51	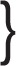	**0.017**
	TT	18 (21.2)	14 (34.1)	Codominant3	5.44	1.35–22.0		
				Dominant	0.84	0.39–1.78		0.64
				Recessive	1.93	0.84–4.42		0.12
				Overdominant^c^	0.24	0.07–0.86		0.013*

miR-146a rs2910164	CC	47 (53.4)	28 (62.2)	Codominant1	0.72	0.33–1.55		0.60
	CG	35 (39.8)	15 (33.3)	Codominant2	0.56	0.11–2.96		
	GG	6 (6.8)	2 (4.4)	Codominant3	0.78	0.14–4.30		
				Dominant^c^	0.70	0.33–1.45		0.33
				Recessive	0.64	0.12–3.28		0.58
				Overdominant	0.76	0.36–1.61		0.47

*SNP, single-nucleotide polymorphism; LRTI, lower respiratory tract infections; TLR, toll-like receptor; OR, odds ratio; CI, confidence interval; NA, not applicable*.

*In the genotype column, the first 2 letters for each SNP represent the WT, the HT and the HM trait, respectively*.

*Codominant1: WT × HT; Codominant2: WT × HM; Codominant3: HT × HM*.

*^a^The ORs, 95% CIs, and P-values were calculated from logistic regression analyses*.

*^b^Bold highlights statistical significance (*p* < 0.05)*.

*^c^This symbol indicates, if applicable, the most probable genetic model of inheritance for the SNP of interest*.

### TLR2 Overexpression in Children With Recurrent Pneumococcal LRTI, and TLR4 Downregulation in Those Carrying the Mutant Homozygous TIRAP 180L Polymorphism

*TLR2* expression on monocytes was significantly higher in LRTI compared with healthy children, although this was a fairly moderate increase (Table 3). Stratified into two age groups (i.e., children <3 years old or children ≥3 years old) this higher *TLR2* expression concerned LRTI children older than 3 years of age (*P* = 0.044) but did not reached significance for younger children (Table 3). Overall, there were no significant differences between the *TLR4* MFI in healthy and LRTI children, even when stratified by age groups (Table 3). We next asked if the cell surface expression of *TLR2* on monocytes was comparable to *TLR4*. *TLR2* MFI were always higher than *TLR4* in both healthy and LRTI children (*P* < 0.001) in all age groups. Based on a simple regression model, we computed correlation coefficients between both TLRs and found that *TLR2* and *TLR4* expressions were strongly correlated in LRTI children (Pearson *r* = 0.64 [95% CI 0.43–0.78], *P* < 0.0001) and moderately in healthy children (Spearman *r* = 0.52 [95% CI 0.17–0.75], *P* = 0.0045). The seven *TLR1, TLR2, TLR4, TLR6*, and *miR-146a* polymorphisms assessed above were not associated with changes of *TLR2* and *TLR4* expression levels at the cell surface of monocytes. Only HM LRTI children for the *TIRAP* variant showed a lower expression of *TLR4* [median: 396.5 (IQR: 376.5–426.5)] compared with homozygous carriers of the wild-type *TIRAP* trait [438.0 (419.0–455.0), *P* = 0.028]. *TLR2* and *TLR4* expression did not vary with gender.

**Table 3:** TLR-2 and TLR-4 expression on monocytes in peripheral blood.

TLR	Age group (years)	*n*	Healthy children	*n*	LRTI children	*P*-value (χ^2^)
			MFI (IQR) [95% CI]		MFI (IQR) [95% CI]	
**TLR2**						

	<3	8	562.5 (528.0–590.5) [514–623]	21	578.0 (542.0–601.0) [543–595]	0.56
	≥3	20	554.5 (530.0–571.0) [533–568]	24	578.0 (540.0–597.3) [541–593]	**0.044**
	All	28	557.0 (530.0–571.8) [534–568]	45	578.0 (540.8–597.0) [563–588]	**0.035**

**TLR4**

	<3	8	438.5 (369.3–468.8) [329–484]	21	423.0 (390.3–448.8) [403–442]	0.91
	≥3	20	427.0 (403.8–439.0) [406–436]	24	439.0 (408.0–476.5) [413–468]	0.12
	All	28	431.0 (400.0–443.8) [406–440]	45	431.5 (404.5–469.5) [415–442]	0.218

*TLR, toll-like receptor; MFI, median fluorescence intensity; IQR, interquartile range; LRTI, lower respiratory tract infections; n, number of children; CI, confidence interval of median*.

### Increased IL-6 Release in LRTI Children After Whole-Blood Stimulation by Spn and TLR4 Ligands

**Figure 1:**
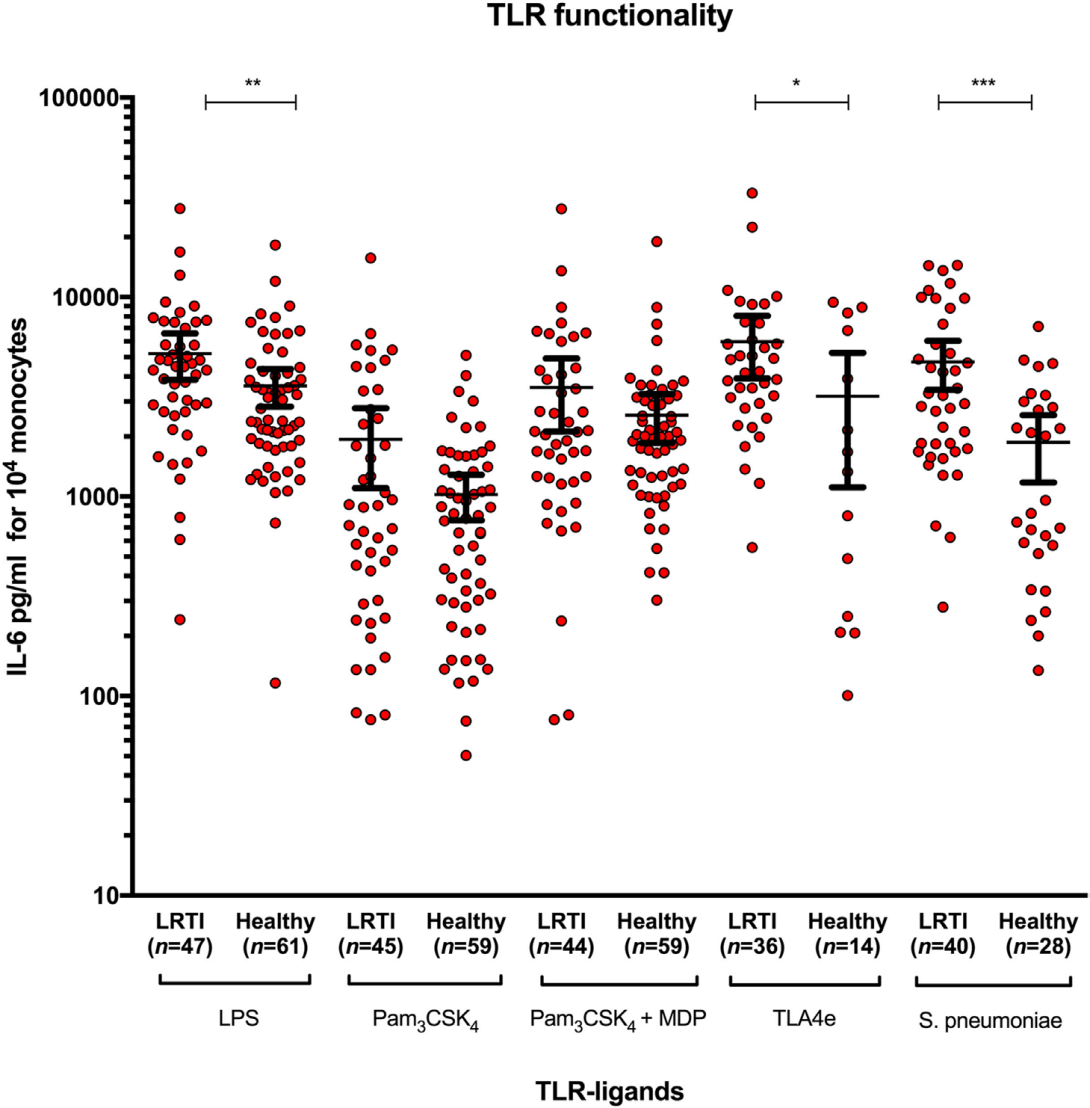
Increased IL-6 release in LRTI children after whole-blood stimulation by *Streptococcus pneumoniae* and *TLR2* and *TLR4* ligands. The diagram shows scatter dot plots of IL-6 secretion in LRTI and healthy children after whole blood stimulation by five TLR ligands. Data are presented as mean and 95% CI (black bars). IL-6 secretion is expressed in picograms per milliliter for 10^4^ monocytes. *P < 0.05; **P < 0.01; and ***P < 0.001 by Mann–Whitney test. Abbreviations: TLR, toll-like receptor; LRTI, lower respiratory tract infections; *n*, number of children; IL-6, interleukin-6.

To study functional response of *TLR2* and *TLR4*, IL-6 secretion was measured as read-out for monocytes activation after stimulation by the corresponding TLR ligands: LPS, Pam_3_CSK_4_, Pam_3_CSK_4_ + MDP, TLA4e, and *Spn* (Figure 1). The amount of IL-6 secreted was significantly higher in LRTI than healthy children when stimulated with LPS (mean [95% CI]: 5,214.6 [3,852.7–6576.5] versus 3,591.4 [2,825.1–4357.7], *P* = 0.0087), TLA4e (5,982.8 [3,913.4–8052.3] versus 3,184.4 [1,112.8–5256.1], *P* = 0.019), and *Spn* (4,732.8 [3,425.0–6040.7] vs 1,870.0 [1,176.1–2563.8], *P* = 0.0004). No significant difference between LRTI and healthy children was observed in the overall amount of IL-6 secreted with Pam_3_CSK_4_ alone (1,937.4 [1,104.1–2770.7] versus 1,023.9 [761.0–1286.9]) or in combination with MDP (3,528.4 [2,126.2–4930.5] versus 2,562.9 [1,861.6–3264.2]). We found no correlation between IL-6 levels and *TLR2* or *TLR4* MFI. In particular, we found no association between IL-6 levels and *TLR2* or *TLR4* MFI after stimulation with seven serial dilutions of *Spn* ranging from 5 × 10^6^ to 78 × 10^3^ cfu/ml.

### Increased Spn-Driven IL-6 Release in LRTI Children Expressing TIRAP Variants

We next investigated whether the susceptibility to LRTI was attributable to functional effects of different SNPs on IL-6 responses (Table S1 in Supplementary Material). In children with the *TLR1* rs4833095 SNP (Figure S1A in Supplementary Material), the IL-6 production was significantly higher in both WT healthy and LRTI children than HM after either Pam_3_CSK_4_ or Pam_3_CSK_4_ + MDP stimulation. Similar results were obtained between HT and HM children. As expected, the synergistic effect of MDP combined with Pam_3_CSK_4_ showed increased levels of IL-6 production in all patients compared with Pam_3_CSK_4_ alone. Overall, there was no significant difference between LRTI and healthy children stimulated with Pam_3_CSK_4_ and Pam_3_CSK_4_ + MDP. Whole blood stimulation with heat-killed *Spn* yielded nearly significant increased IL-6 production in WT LRTI children compared with healthy children (*P* = 0.0502).

Regarding *TLR1* rs5743618 SNP (Figure S1B in Supplementary Material), more IL-6 was produced in WT than in HM both in LRTI and healthy children after Pam_3_CSK_4_ stimulation, and between WT and HT healthy children. Pam_3_CSK_4_ + MDP stimulation induced increased IL-6 levels only in WT healthy children compared with HM. Similarly to *TLR1* rs4833095 SNP, we noted for rs5743618 SNP a trend toward an increased IL-6 production after *Spn* stimulation in WT LRTI children compared with healthy children (*P* = 0.07). The difference was significant between HM LRTI and healthy children.

After stimulation with *Spn*, we observed increased IL-6 levels in WT LRTI compared with WT healthy children for *TLR2* rs5743708, *TLR4* rs4986790, and *TLR4* rs4986791 (Figures S2A and 3A,B in Supplementary Material, respectively). When stimulated by Pam_3_CSK_4_, WT LRTI children with *TLR6* rs5743810 SNP produced more IL-6 than HT and HM patients (Figure S2B in Supplementary Material). In addition, IL-6 tended to be higher in WT LRTI than in WT healthy children (*P* = 0.052). Pam_3_CSK_4_ + MDP showed only a difference between WT and HT LRTI children. Again, WT LRTI children produced more IL-6 than healthy children when challenged by *Spn* (Figure S2B in Supplementary Material).

**Figure 2:**
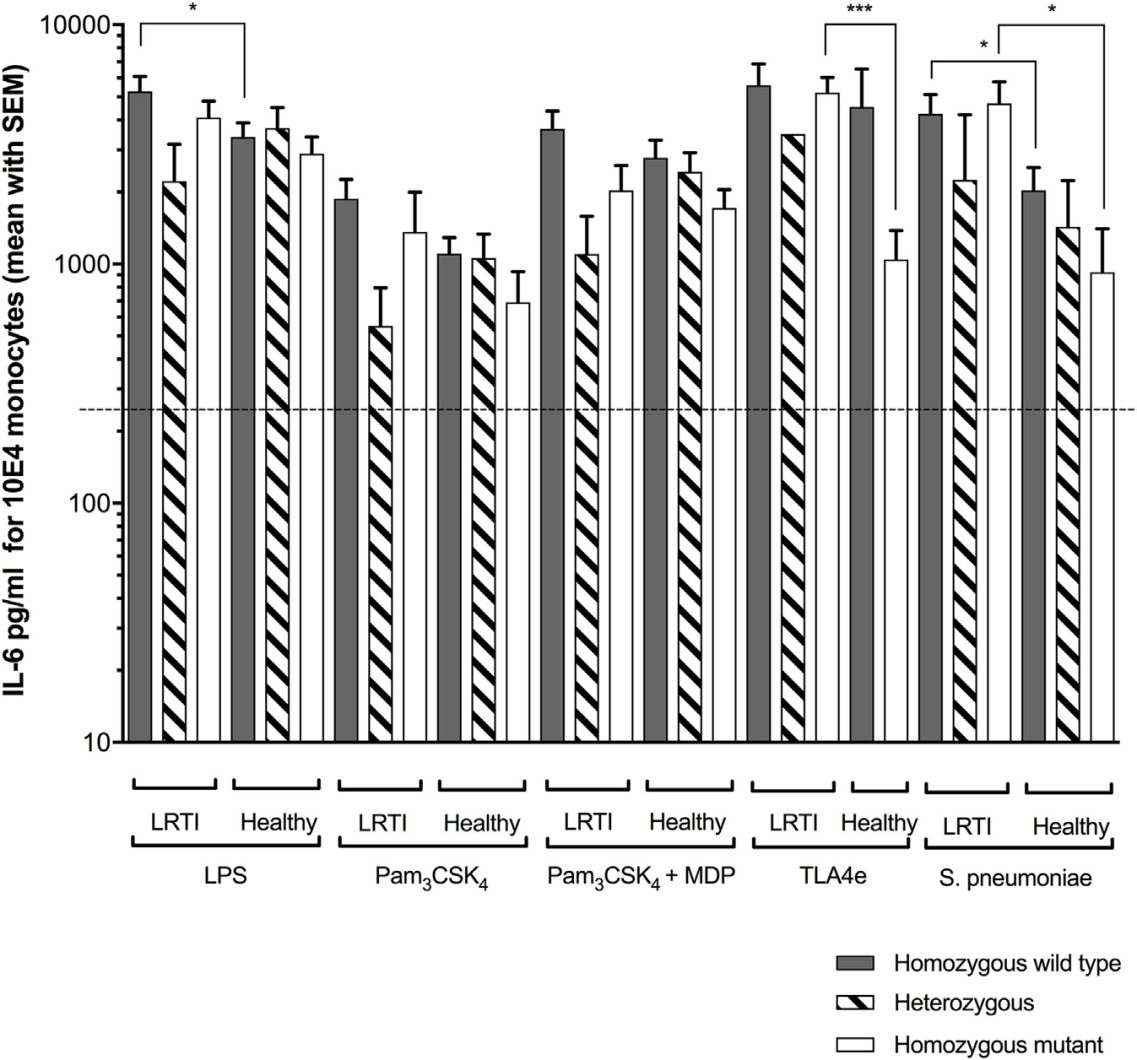
Increased *Streptococcus pneumoniae* (*Spn*)-driven IL-6 release in LRTI children expressing *TIRAP* variants. This figure shows the impact of TIRAP rs8177374 polymorphism on monocyte release of IL-6 following *ex vivo* whole blood stimulation in LRTI and healthy children by either *E. coli* K12 LPS (200 ng/ml final), Pam3CSK4 (10 µg/ml final), Pam3CSK4 (20 µg/ml final) + MDP (200 µg/ml final), TLA4e/AF04 (400 ng/ml final), or heat-killed *Spn* (1 × 10^8^ cfu/ml). Gray histograms represent homozygous wild-type individuals, dashed histograms represent heterozygous, and white histograms represent homozygous mutant. Upper borders of histograms denote mean and solid horizontal lines denote SEM. The dashed lines represent the limit detection threshold (250 pg/ml). IL-6 secretion is expressed in picograms per milliliter for 10^4^ monocytes. Number of children per histogram is provided in Table 4. *P < 0.05; **P < 0.01; ***P < 0.001; and ****P < 0.0001 by Mann–Whitney test. Abbreviations: LRTI, lower respiratory tract infections; IL-6, interleukin-6.

Regarding *TIRAP* rs8177374 SNP (Figure 2), we found that LPS produced more IL-6 in WT LRTI than in healthy children (*P* = 0.011). Furthermore, TLA4e yielded more IL-6 in HM LRTI children (*P* = 0.0002). In addition, *Spn* produced more IL-6 in WT (*P* = 0.031) or HM LRTI children (*P* = 0.019) than healthy children.

Finally, with miR-146a rs2910164 SNP (Figure S4 in Supplementary Material), we found that IL-6 production was increased in HT LRTI children compared with WT LRTI children when stimulated by either Pam_3_CSK_4_ or Pam_3_CSK_4_ + MDP. We observed similar results in healthy children stimulated by Pam_3_CSK_4_ + MDP. Stimulation by TLA4e and *Spn* yielded more IL-6 production in HT LRTI than in HT healthy children. More IL-6 was produced in response to *Spn* in WT LRTI than in healthy children.

### TLR4 Asp299Gly/Thr399Ile Haplotype Does Not Affect IL-6 Release in LRTI Children

**Figure 3:**
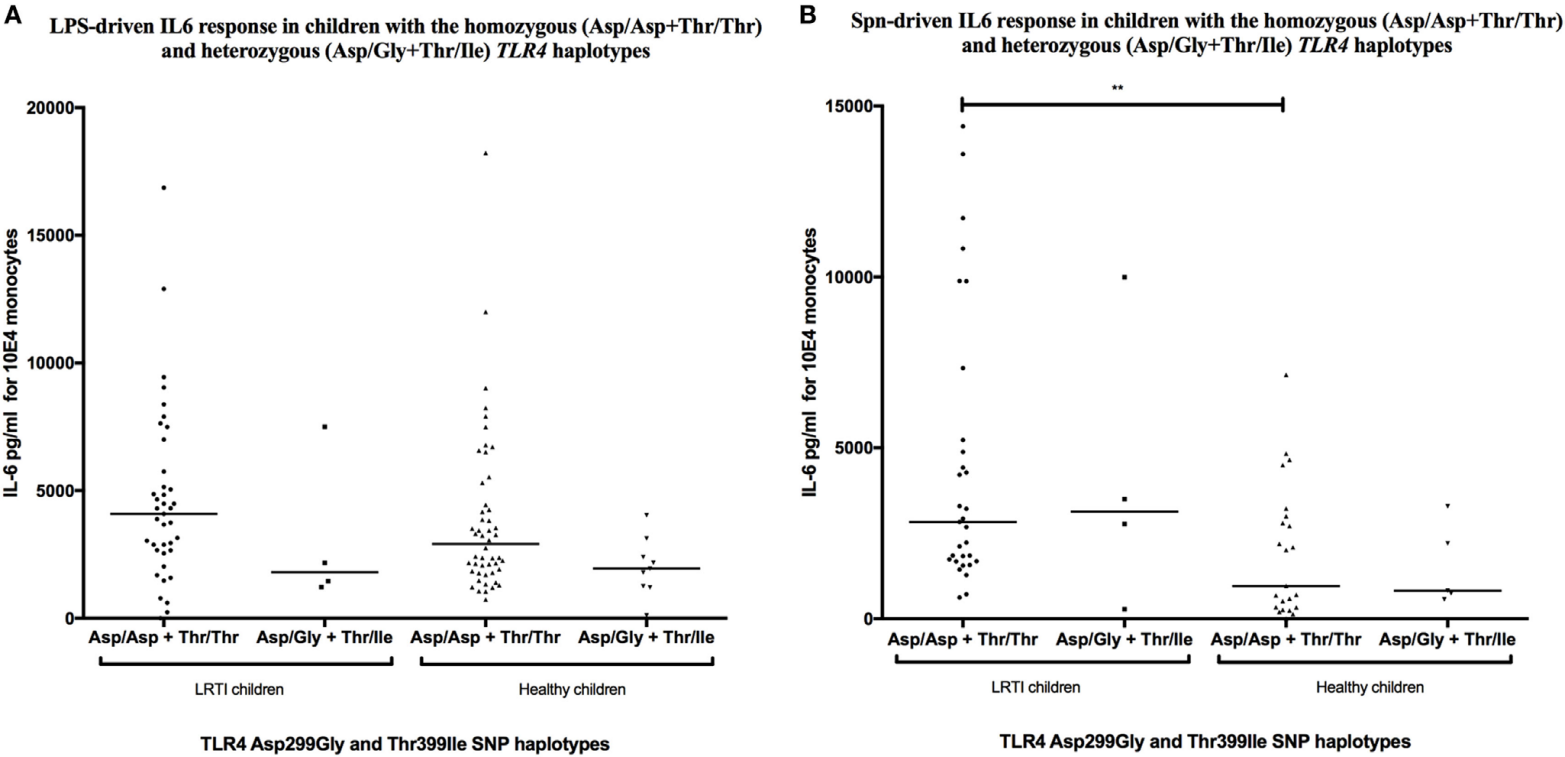
*TLR4* Asp299Gly/Thr399Ile haplotype does not influence IL-6 release in LRTI children. This figure shows scatter dot plots of IL-6 secretion in TLR4 Asp299Gly and Thr399Ile haplotypes co-segregation after **(A)** LPS or **(B)** *Streptococcus pneumoniae* (*Spn*) stimulation. IL-6 secretion is expressed in picograms per milliliter for 10^4^ monocytes. Solid line denotes mean. *P < 0.05; **P < 0.01; ***P < 0.001; and ****P < 0.0001 by Mann–Whitney test. Abbreviations: TLR, toll-like receptor; LRTI, lower respiratory tract infections; IL-6, interleukin-6.

Consistent with the 98% co-segregation level described in Caucasian populations (35), *TLR4* Asp299Gly and Thr399Ile SNPs were, in our study, co-segregated in 44 of 45 LRTI children (97.8%) and in 86 of 87 healthy children (98.9%). IL-6 production was not influenced by the *TLR4* haplotype with LPS or *Spn* stimulation. The IL-6 production was indeed similar between LRTI children with the double-homozygous wild-type haplotype (AA + CC) and the heterozygous haplotype (AG + CT) (Figure 3). Only healthy double WT children showed decreased levels of IL-6 compared with LRTI children when stimulated by *Spn* (mean [95% CI]: 4,444 [2,988–5,901] versus 1,917 [1,081–2,753], *P* = 0.0045).

### Combined Nucleotide Polymorphisms in TLR1 or TLR6 and TIRAP Are Associated With a Decreased TLR2-Ligand-Driven IL-6 Release in LRTI Children

**Figure 4:**
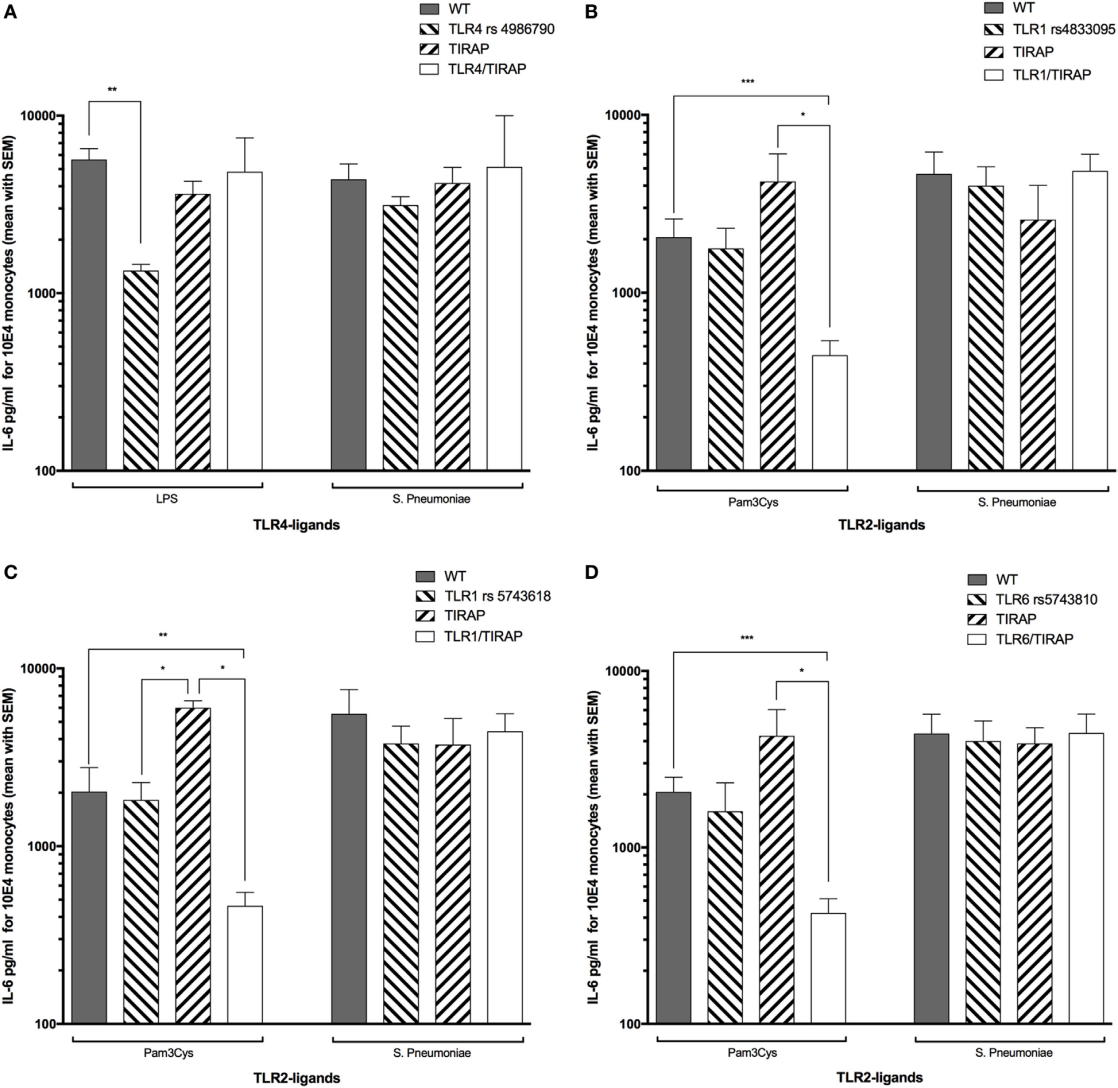
Combined nucleotide polymorphisms in *TLR1* or *TLR6* and *TIRAP* are associated with a decreased *TLR2* ligand-driven IL-6 release in LRTI children. This figure shows the impact of *TIRAP* rs8177374, *TLR1* rs4833095, *TLR1* rs5743618, *TLR4* rs4986790, or *TLR6* rs5743810 polymorphisms or their combinations on monocyte release of IL-6 following *ex vivo* whole blood stimulation by LPS, Pam3Cys, or *Streptococcus pneumoniae* (*Spn*). Values are shown as mean with SEM. Number of children per histogram may differ from those in Table 4 (due to unavailable IL-6 values). **(A)** Number of patients in each group: wild type (WT) = 20, *TIRAP* [heterozygous (HT) and homozygous mutant (HM) reported together] = 15, and *TLR4*/*TIRAP* (HT and HM reported together) = 2, *TLR4* = 2. **(B)** WT = 8, *TIRAP* (HT and HM reported together) = 3, *TLR1* rs4833095/*TIRAP* (HT and HM reported together) = 12, and *TLR1* rs4833095 (HT and HM reported together) = 14. **(C)** Controls = 6, *TIRAP* (HT and HM reported together) = 2, *TLR1* rs574361*8*/*TIRAP* (HT and HM reported together) = 13, and *TLR1* rs5743618 (HT and HM reported together) = 16. **(D)** Controls = 13, *TIRAP* (HT and HM reported together) = 3, *TLR6*/*TIRAP* (HT and HM reported together) = 12, and *TLR6* (HT and HM reported together) = 9. Gray histograms represent homozygous wild-type individuals, dashed histograms represent heterozygous, and white histograms represent homozygous mutant. Upper borders of histograms denote mean, and solid horizontal lines denote SEM. IL-6 secretion is expressed in picograms per milliliter for 10^4^ monocytes. *P < 0.05; **P < 0.01; and ***P < 0.001 by Mann–Whitney test. Abbreviations: TLR, toll-like receptor; WT, wild type; IL-6, interleukin-6.

Combination of *TLR4* and *TIRAP* SNPs has been described to be associated with low circulating levels of TNF-α and IL-6 in adult patients with ventilator-associated pneumonia following 1 ng/ml LPS-stimulation (36). We investigated in our study the frequency of this double mutation in LRTI children (genotypes distribution are summarized in Table 4) and whether it was similarly associated with low levels of IL-6. In our study, IL-6 circulating levels were not different in children with or without combined mutations in *TLR4* and *TIRAP* genes after stimulation by 1.56 ng/ml LPS or 62,500 cfu of *Spn* (Figure 4A). After stimulation by Pam_3_CSK_4_, children with combined mutations in *TLR1* rs4833095 or *TLR1* rs5743618 and *TIRAP* exhibited a deficient IL-6 secretion compared with WT children (*P* < 0.001 and *P* < 0.01, respectively) or children carriers of only *TLR1* or *TIRAP* polymorphisms (*P* < 0.05, Figures 4B,C). Similar results were found for combined mutations in *TLR6* rs5743810 (*P* < 0.001) and *TIRAP* (*P* < 0.05, Figure 4D). Given that no LRTI children had *TLR2* homozygous mutations, we were not able to similarly assess combination of *TLR2* and *TIRAP* SNPs. We found no association with these combined mutations and IL-6 levels when monocytes from LRTI children were stimulated with *Spn*.

**Table 4:** Distribution of TLR1, 2, 4, or 6 and TIRAP genotypes in LRTI children.

SNPs	Genotypes	TIRAP		
		Homozygous wild type, *n* (%)	Heterozygous, *n* (%)	Homozygous mutant, *n* (%)
TLR4 rs4986790	Homozygous wild type	22 (53.7%)	2 (4.9%)	13 (31.7%)
	Heterozygous	2 (4.9%)	1 (2.4%)	1 (2.4%)
	Homozygous mutant	–	–	–

TLR1 rs4833095	Homozygous wild type	9 (22.5%)	1 (2.5%)	2 (5%)
	Heterozygous	12 (30%)	1 (2.5%)	4 (10%)
	Homozygous mutant	3 (7.5%)	1 (2.5%)	7 (17.5%)

TLR1 rs5743618	Homozygous wild type	7 (17.1%)	–	2 (4.9%)
	Heterozygous	12 (29.3%)	2 (4.9%)	3 (7.3%)
	Homozygous mutant	5 (12.2%)	1 (2.4%)	9 (22%)

TLR2 rs5743708	Homozygous wild type	23 (56.1%)	3 (7.3%)	14 (34.1%)
	Heterozygous	1 (2.4%)	–	–
	Homozygous mutant	–	–	–

TLR6 rs5743810	Homozygous wild type	15 (36.6%)	1 (2.4%)	2 (4.9%)
	Heterozygous	6 (14.6%)	2 (4.9%)	8 (19.5%)
	Homozygous mutant	3 (7.3%)	–	4 (9.8%)

*TLR, toll-like receptor; TIRAP, toll-interleukin 1 receptor domain-containing adaptor protein; SNP, single-nucleotide polymorphism; LRTI, lower respiratory tract infection; n, number of children*.

## Discussion

To the best of our knowledge, this is the first study to show an association between SNP in *TIRAP* and increased susceptibility to recurrent pneumococcal LRTI in children. Previous studies have reported that common genetic variations in TLRs affect host susceptibility to infectious diseases (37–39). In particular, some authors found a relationship between IPD and SNPs in TLRs (40) or Toll-IL1R (TIR) signaling pathway proteins (41), but none of them studied specifically *TIRAP* genes. In our study, the key finding was that the *TIRAP S180L* heterozygous trait contributes to protection to recurrent pneumococcal LRTI in children, whereas the 180L homozygous mutant trait, alone or in combination with *TLR1* N248S, *TLR1* I602S or *TLR6* S249P polymorphisms, contributes to increased susceptibility by modulating the inflammatory response. Children carrying the mutant homozygous *TIRAP* 180L polymorphism were indeed more likely to have recurrent pneumococcal LRTI, while carrying the heterozygous S180L polymorphism decreased the likelihood to be in this category.

Interestingly, similar results have been described in adult studies where *TIRAP* polymorphism in its heterozygous S180L form conferred protection against primary immune deficiency, or septic shock (42). It decreased the risk of an excessive inflammatory response by attenuating *TLR2* and *TLR4* signal transduction through the impairment of cytokine responses and NF-κB activation following stimulation with *TLR2* and *TLR4* ligands (36, 43, 44). Our results extend these previous adult findings to pediatrics. Likewise, the same pattern of *TIRAP* associations was observed in other Gram-positive or Gram-negative bacterial infections, bacteremia, malaria, and tuberculosis in more than 6,100 individuals (44). Since *TIRAP* is essential and restricted to the *TLR2*- (with *TLR1* and *TLR6* as co-receptors) and *TLR4*-mediated MyD88-dependent signaling pathway (45), it is possible that a genetic variation at this central position explains the variable susceptibility of the host’s immune response to various infectious diseases. In accordance with previous human studies (19, 46), we observed no direct associations between susceptibility to pneumococcal LRTI and the other SNPs assessed in our study. Our results support the interpretation that the *TIRAP* S180L polymorphism might affect directly the clinical course of recurrent pneumococcal LRTI in children whereas SNPs in *TRL2* or *TLR4* are not directly associated with an increased risk of IPD. They are in line with those from other studies that have shown that individuals homozygous for the *TIRAP* 180L variant have an increased risk of infection, in particular sepsis (42) and invasive *Haemophilus influenzae* serotype b (Hib) infection in immunized children (47).

Controversial results have been reported regarding the impact of the *TLR2* and *TLR4* polymorphisms on the expression levels. First, we observed that *TLR2* was significantly up-regulated in LRTI compared with healthy children, independently of any SNPs that we tested. In line with our results, *TLR2* upregulation on monocytes during the clinical course of infections has already been observed in adults and seemed even to be a possible predictor of recurrence in patients with bacterial infectious diseases (48). The fact that this upregulation was particularly marked in LRTI children above 3 years old in our study might reflect their sustained recurrent episodes of pneumococcal infections over years. Second, conflicting results exist on whether *TLR4* polymorphisms may impair trafficking and cell surface expression of *TLR4* (11, 13, 49). In our study and others (50, 51), it was observed that *TLR4* was only down-regulated in *TIRAP* homozygous mutant carriers. This reduced level of *TLR4* expression might be one of the factors underlying the susceptibility to pneumococcal recurrent LRTI in this subpopulation. Future studies are required to further examine this question.

In addition to genetic association between TLRs polymorphisms and cell surface expression of *TLR2* and *TLR4*, we also indirectly investigated the integrity of the children’s *TLR2* and *TLR4* signaling pathways by investigating various TLR ligands-driven IL-6 responses. With the use of a whole blood stimulation assay, we observed that the overall *Spn*-, LPS- and TLA4e-driven IL-6 levels were higher in children with recurrent pneumococcal LRTI than in healthy children. However, according to other studies (52, 53), we observed that the Asp299Gly/Thr399Ile haplotype had no different IL-6 production after stimulation with LPS and *Spn*. By stratifying the children according to the various SNPs studied, we found that *TIRAP* was the gene most commonly involved in these IL-6 levels differences. Although restricted by unavailable data in some SNPs subpopulations, especially HM genotypes, we found that *Spn*-driven IL-6 levels were specifically increased in LRTI children compared with healthy children for all SNPs studied. This was especially true in both WT and HM subpopulations with *TIRAP* polymorphisms.

In addition, we observed that other specific TLR ligands—but not *Spn*—yielded a significant reduction in IL-6 levels in HM children for the *TLR1, TLR6* and *TIRAP* genes, but without a difference between the healthy and LRTI children. This reduction was even more obvious when individuals were assessed for combined double mutations with *TIRAP*. Unlike Kumpf et al. who showed that combination of *TLR4* and *TIRAP* SNPs was associated with low circulating levels of IL-6 in adult patients with ventilator-associated pneumonia (36), we observed instead significantly reduced IL-6 levels in *TLR1* or *TLR6* and *TIRAP* double-mutant children following Pam_3_CSK_4_ stimulation. This difference might be related to the nature of the bacteria studied. Indeed, whereas the patients studied by Kumpf et al. developed pneumonia predominantly caused by Gram-negative bacteria, known to be specifically recognized by *TLR4*, the children in our study were supposedly infected by Gram-positive *Spn* recognized by both *TLR2* (and *TLR1* and *TLR6* as co-receptors) and *TLR4*. The fact that Pam_3_CSK_4_, a specific *TLR2* ligand, was unable to induce potent IL-6 production in double-mutated LRTI children (*TIRAP* 180Leu/*TLR1* 248S or *TIRAP* 180Leu/*TLR1* 602S or *TIRAP* 180Leu/*TLR6* 249P) might reflect a defect within the *TLR2* or *TLR1* and *TLR6* co-receptors. On the other hand, given that *Spn*-induced IL-6 levels were preserved in double-mutant LRTI children might supposedly reflect the role played by the intact *TLR4* pathway. This redundant *TLR2*–*TLR4* recognition system might serve, at least in part when *TIRAP* is functional, as a rescue system to prevent the escape of some microorganisms from the innate immune system. However, these findings raised the possibility that the redundant signaling cascades using *TLR2* and *TLR4* paths may be overwhelmed in pneumococcal infections if *TIRAP* becomes less functional through homozygote mutations. This may explain why in our study, only children with *TIRAP* mutations showed abnormal IL-6 levels when compared with healthy children after a *Spn* challenge. Taken together, these results showed that the TLR signaling system needs to be tightly regulated to constantly balance between activation and inhibition to avoid inappropriate inflammatory responses (54). A plausible way by which *TIRAP* polymorphisms may explain varying susceptibilities to *Spn* in children might rely on an imbalance of downstream proinflammatory cytokines levels. Although the exact mechanism by which this imbalance leads in the end to pneumonia extends beyond the scope of this study and needs further investigations. However, whereas increased TLR-driven pro-inflammatory responses play a pivotal role in host defense against invading pathogens, altered TLR function might contribute to destructive inflammatory damages and increased susceptibility to infection. This hypothesis is supported by a study performed by Ferwarda et al. These authors observed that a moderately increased cytokine release during the primary stage of infection, as would be expected in heterozygous S180L individuals, is likely beneficial for the outcome of the infection and the early clearance of bacteria. However, if the cytokine release is reaching a certain threshold leading to systemic inflammatory effects, which was suggested to take place in homozygous 180L individuals, then this could ultimately prove deleterious to the host (42). In our study, LRTI children homozygous for the *TIRAP* 180L mutant trait appeared to be deprived of the attenuating effect provided by the S180L and more prone to excessive IL-6 levels production in response to *Spn* compared with healthy controls. According to Khor et al., this finding was consistent with increasing evidence that an excessive host inflammatory status might render individuals more susceptible to infectious diseases (44). However, other microbial, environmental, genetic and immune factors may also contribute to the susceptibility for infection, as children’s infection rates usually spontaneously improve after the age of 5.

Our study has some limitations. First, LRTI is defined clinically according to the WHO definitions. Because up to 50% of children with pneumonia have no bacterial proof despite thorough investigations (55), obtaining samples confirming the presence of *Spn* for each child would be difficult and probably unethical. However, local and international epidemiological studies recognize *Spn* as the most prevalent cause of LRTIs in young children (23). This limitation was already addressed earlier (14).

Second, we did not assess other adaptor molecules that have been described to be associated with an increased susceptibility to invasive bacterial infections (18, 40, 56–61). It would be interesting to compare the influence of these molecules to *TIRAP* polymorphism on recurrent pneumococcal LRTI in children in future studies. However, these defects can be a diagnostic challenge because the affected patients present narrow susceptibility ranges for invasive pyogenic bacterial infections (20), and their routine immunological screening is often normal.

Third, because blood sampling was performed only once in healthy children, it is possible that normal interindividual variations were missed. Since our results were obtained from a modest number of patients, further studies with larger population sizes and investigating defects along the TLR2 and 4 pathways in children with pneumococcal LRTI would be valuable. Moreover, although the children were shown to have no total Ig deficiency (14), we cannot totally exclude that some children had an unrecognized underlying rare immune deficiency.

Fourth, *TLR4* requires the involvement of the MD-2 glycoprotein and CD14 as co-receptors on its extracellular domain to form a complex able to recognize and bind LPS, with the help of LPS binding protein (62). Because we did not assess polymorphism in these molecules, we cannot exclude that other SNPs may affect susceptibility to recurrent LRTI. To date, studies on MD-2 or CD14 polymorphisms and association to PID remain rare (40, 63).

Sixth, the quality of antibodies could have influenced the MFI results. However, fluorochrome-matched monoclonal anti-TLR2 and anti-TLR4 were purchased from the same company and prepared following manufacturer’s instructions at optimal dilution to ensure reliability of the results. These commercially available antibodies are expected to have undergone extensive quality control programs to meet the manufacturer’s strict release criteria. Median fluorescence intensity were chosen as a read-out instead of mean fluorescence intensity because median is generally considered a much better statistical approach in that median is less influenced by skew or outlier events.

Finally, we did not assess *TLR1* and *TLR6* expression at the cell surface of the monocytes. Given our results showing the impact of related SNPs on these TLRs, further analysis of SNPs in these TLRs would be interesting to fully understand these pathways and clarify their precise role in PID.

## Restrictions Apply to the Datasets

The datasets for this manuscript are not publicly available to avoid patients’ identification and to preserve anonymization. Requests to access the anonymized datasets should be directed to Dr. Johan Siebert at johan.siebert@hcuge.ch.

## Ethics Statement

The study was approved by the institutional ethics committee, and written consent was obtained from all parents or legal guardians. It was conducted in accordance with the principles of the Declaration of Helsinki, the standards of Good Clinical Practice, and Swiss regulatory requirements.

## Author Contributions

Conceptualization: JS and KP-B; methodology: JS and KP-B; investigation: JS, KP-B, and SG; formal analysis: JS and CV; validation: CG and LH; writing—original draft: JS; visualization: JS; writing—review and editing: JS, LH, C-AS, and KP-B; funding acquisition: KP-B; resources: CG, LH, and C-AS; supervision: C-AS and KP-B. All authors discussed the results and commented on the manuscript. All authors have contributed to, seen and approved the final, submitted version of the manuscript, and had full access to all of the data (including statistical reports and tables) in the study and can take responsibility for the integrity of the data and the accuracy of the data analysis. The corresponding author confirms that he had full access to participant’s data and endorsed the final responsibility for the submission, and affirms that the manuscript is an honest, accurate, and transparent account of the study being reported; that no important aspects of the study have been omitted; and that any discrepancies from the study as planned have been explained.

## Conflict of Interest Statement

The authors declare that the research was conducted in the absence of any commercial or financial relationships that could be construed as a potential conflict of interest.

## Abbreviations

SNP, single-nucleotide polymorphism; LRTI, lower respiratory tract infections; *Spn, Streptococcus pneumoniae*; TLR, toll-like receptor; TIRAP, toll-interleukin 1 receptor domain-containing adaptor protein; CAP, community-acquired pneumonia; IPD, invasive pneumococcal disease; MFI, median fluorescence intensity; WT, wild type; HT, heterozygous; HM, homozygous.
