# Supplementary material for: Toll-Interleukin 1 Receptor Domain-Containing Adaptor Protein 180L Single-Nucleotide Polymorphism Is Associated With Susceptibility to Recurrent Pneumococcal Lower Respiratory Tract Infections in Children
Source: Front Immunol. 2018 Aug 7;9:1780. doi: 10.3389/fimmu.2018.01780 (PMC6090034; doi:10.3389/fimmu.2018.01780)
Supplement: Supplementary file 1 [file table_1.docx]

**Supplementary Table 1.**

| MEAN TLR_ligands_-INDUCED IL-6 SECRETION ACCORDING TO SNPs | | | | | | | |
| --- | --- | --- | --- | --- | --- | --- | --- |
|  | | **TLR-ligands** | | | | | |
| **SNP** | | **Pam_3_CSK_4_**  Mean [95%CI] | **Pam_3_CSK_4_+MDP**  Mean [95%CI] | **LPS**  Mean [95%CI] | | **TLA4e**  Mean [95%CI] | **Spn**  Mean [95%CI] |
| **TLR1 rs4833095** | | | | | | | |
| LRTI | CC (*n*=6/10/12) | 2649 [1200-4097] | 3873 [1174-6573] | |  |  | 4035 [1434-6637] |
|  | CT (*n*=19/19/19) | 1642 [777.8-2506] | 3294 [1975-4612] | |  |  | 4542 [2524-6561] |
|  | TT (*n*=11/11/7) | 356.9 [148-565.9] | 1483 [623.4-2343] | |  |  | 4620 [1075-8165] |
| Healthy | CC (*n*=20/20/12) | 1622 [986.7-2257] | 3480 [1596-5364] | |  |  | 1891 [511.2-3270] |
|  | CT (*n*=23/22/10) | 901 [620.1-1182] | 2504 [1726-3282] | |  |  | 2032 [961.7-3101] |
|  | TT (*n*=13/14/5) | 351.4 [180.8-522.1] | 1394 [872.4-1915] | |  |  | 1744 [0-3949] |
| **TLR1 rs5743618** | | | | | | | |
| LRTI | GG (*n*=8/7/7) | 3024 [1008-5040] | 4605 [571.5-8639] | |  |  | 5017 [1375-8659] |
|  | GT (*n*=16/16/16) | 1580 [664.3-2496] | 2773 [1585-3961] | |  |  | 3231 [1819-4642] |
|  | TT (*n*=17/17/13) | 869.7 [238-1501] | 2413 [1125-3701] | |  |  | 5553 [2609-8497] |
| Healthy | GG (*n*=12/12/6) | 1897 [1189-2606] | 3129 [1901-4356] | |  |  | 2152 [375.9-3927] |
|  | GT (*n*=23/23/11) | 986.6 [519.1-1454] | 3097 [1429-4766] | |  |  | 1953 [442.3-21484] |
|  | TT (*n*=21/21/10) | 584.1 [335.9-832.4] | 1686 [1187-2186] | |  |  | 1733 [685.4-2781] |
| **TLR2 rs5743708** | | | | | | | |
| LRTI | GG (*n*=39/38/34) | 1526 [939.5] | 2828 [1932-3724] | |  |  | 4194 [2877-5510] |
|  | GA (*n*=2/2/2) | 2364 [0-28568] | 5084 [0-53624] | |  |  | 8200 [0-52931] |
| Healthy | GG (*n*=52/52/27) | 1046 [751.2-1341] | 2614 [1821-3406] | |  |  | 1916 [1201-2630] |
|  | GA (*n*=4/4) | 829 [0-1797] | 2073 [0-4148] | |  |  |  |
| **TLR6 rs5743810** | | | | | | | |
| LRTI | TT (*n*=19/18/17) | 2315 [1374-3257] | 3919 [2192-5646] | |  |  | 4774 [2590-6959] |
|  | TC (*n*=15/15/12) | 1088 [124.9-2050] | 2018 [918.2-3117] | |  |  | 3547 [1413-5680] |
|  | CC (*n*=7/7/7) | 563.8 [2.81-1125] | 2403 [656.4-4150] | |  |  | 5038 [1300-8777] |
| Healthy | TT (*n*=30/30/17) | 1296 [825.3-1766] | 3008 [1653-4362] | |  |  | 1951 [938.1-2964] |
|  | TC (*n*=21/21/9) | 790.6 [521.7-1060] | 2210 [1753-2668] | |  |  | 2024 [803.4-3245] |
|  | CC (*n*=5/5/1) | 449.4 [0-907.6] | 1511 [384.3-2638] | |  |  | 340.7 [NA] |
| **TLR4 rs4986790** | | | | | | | |
| LRTI | AA (*n*=38/29/32) |  |  | | 4768 [3687-5849] | 5472 [3858-7086] | 4135 [0-10726] |
|  | AG (*n*=4/3/4) |  |  | | 3088 [0-7814] | 3108 [0-12386] | 2004 [1148-2861] |
| Healthy | AA (*n*=50/11/22) |  |  | | 3855 [2947-4762] | 3635 [1047-6224] | 1526 [58.9-2993] |
|  | AG (*n*=9/3/5) |  |  | | 2009 [1134-2884] | 1531 [0-6634] |  |
|  | GG (*n*=1/0/0) |  |  | | 3601 [NA] | 4452 [3043-5860] |  |
| **TLR4 rs4986791** | | | | | | | |
| LRTI | CC (*n*=39/29/32) |  |  | | 4676 [3580-5773] | 5363 [3717-7010] | 4392 [2980-5804] |
|  | CT (*n*=4/3/4) |  |  | | 3088 [0-7814] | 3108 [0-12386] | 4135 [0-10726] |
| Healthy | CC (*n*=50/11/22) |  |  | | 3855 [2947-4762] | 3635 [1047-6224) | 2004 [1148-2861] |
|  | CT (*n*=9/3/5) |  |  | | 2009 [1134-2884] | 1531 [0-6634] | 1526 [58.9-2993] |
| **TIRAP rs8177374** | | | | | | | |
| LRTI | CC (*n*=22/22/22/17/19) | 1877 [1078-2676] | 3677 [2250-5105] | | 5268 [3551-6985] | 5599 [2897-8302] | 4244 [2408-6081] |
|  | CT (*n*=3/3/3/1/2) | 551.8 [0-1603] | 1101 [0-3179] | | 2223 [0-6296] | 3499 [NA] | 2244 [0-27213] |
|  | TT (*n*=14/12/11/10/11) | 1364 [0-2760] | 2028 [786.9-3270] | | 4098 [2569-5628] | 5207 [3354-7061] | 4696 [2286-7106] |
| Healthy | CC (*n*=39/38/39/5/16) | 1105 [726.1-1483] | 2779 [1740-3819] | | 3394 [2384-4404] | 4539 [0-10112]] | 2031 [964.6-3098] |
|  | CT (*n*=8/8/8/0/3) | 1059 [412.1-1706] | 2434 [1292-3575] | | 3707 [1799-5614] |  | 1432 [0-4295] |
|  | TT (*n*=10/8/8/6/5) | 692.1 [133.6-1251] | 1714 [927.4-2501] | | 2897 [1759-4035] | 1045 [183.9-1907] | 924 [0-2259] |
| **miR146a rs2910164** | | | | | | | |
| LRTI | CC (*n*=28/25/24/17/21) | 1155 [509-1801] | 2000 [1220-2779] | | 3737 [2837-4637] | 3929 [2841-5018] | 3736 [2121-5351] |
|  | CG (*n*=13/14/14/13/13) | 2458 [1241-3675] | 4734 [2672-6796] | | 6166 [3455-8876] | 6713 [3301-10125] | 5337 [2792-7881] |
|  | GG (*n*=2/2/2/2/2) | 482.2 [0-3485] | 1682 [1515-1849] | | 6763 [0-35673] | 6973 [0-55690] | 5579 [0-60238] |
| Healthy | CC (*n*=31/29/29/8/16) | 887.8 [491.9-1284] | 2457 [1093-3821] | | 3576 [2196-4955] | 4658 [1243-8073] | 1880 [788.1-2972] |
|  | CG (*n*=25/24/24/6/11) | 1193 [750.3-1636] | 2692 [2058-3327] | | 3611 [2807-4415] | 1220 [0-2708] | 1967 [975.1-2960] |
|  | GG (*n*=3/3/3/0/0) | 1114 [0-3406] | 2780 [0-7192] | | 3981 [0-10809] |  |  |

SNP, Single Nucleotide Polymorphism; LRTI, Lower Respiratory Tract Infections; TLR, toll-like receptor; CI, confidence interval;

NA, not applicable; n, number of patients for each genotype and for each TLR-ligand respectively.

Mean IL-6 are given in pg/ml for 10^4^ monocytes.
