# Supplementary material for: Toll-Interleukin 1 Receptor Domain-Containing Adaptor Protein 180L Single-Nucleotide Polymorphism Is Associated With Susceptibility to Recurrent Pneumococcal Lower Respiratory Tract Infections in Children
Source: Front Immunol. 2018 Aug 7;9:1780. doi: 10.3389/fimmu.2018.01780 (PMC6090034; doi:10.3389/fimmu.2018.01780)
Supplement: Supplementary file 5 [file data_sheet_4.docx]

**Supplementary Figure 4**

The figure shows the impact of miR-164a rs2910164 polymorphism on monocyte release of IL-6 following ex-vivo whole blood stimulation by either E. Coli K12 LPS (200ng/ml final), Pam_3_CSK_4_ (10μg/ml final), Pam_3_CSK_4_ (20μg/ml final) + MDP (200μg/ml final), TLA4e/AF04 (400ng/ml final), or heat-killed Spn (1×10^8^ cfu/ml). Gray histograms represent homozygous wild type individuals, dashed histograms represent heterozygous, and white histograms represents homozygous mutant. Upper borders of histograms denote mean and solid horizontal lines denote standard error on the mean (SEM). The dashed lines represent the limit detection threshold (250 pg/ml). IL-6 secretion is expressed in pg/ml for 10^4^ monocytes. Number of children per histogram is provided in Table 4. * P < 0.05; ** P < 0.01; *** P < 0.001; **** P < 0.0001 by Mann-Whitney test. LRTI: Lower Respiratory Tract Infections.
